# Potential Utilization of APOBEC3-Mediated Mutagenesis for an HIV-1 Functional Cure

**DOI:** 10.3389/fmicb.2021.686357

**Published:** 2021-06-15

**Authors:** Terumasa Ikeda, Yuan Yue, Ryo Shimizu, Hesham Nasser

**Affiliations:** ^1^Division of Molecular Virology and Genetics, Joint Research Center for Human Retrovirus Infection, Kumamoto University, Kumamoto, Japan; ^2^Graduate School of Medical Sciences, Kumamoto University, Kumamoto, Japan

**Keywords:** APOBEC3-mediated mutagenesis, genetic factors, A3 expression, A3-interacting proteins, adaptive immunity, Vif inhibitors

## Abstract

The introduction of combination antiretroviral therapy (cART) has managed to control the replication of human immunodeficiency virus type 1 (HIV-1) in infected patients. However, a complete HIV-1 cure, including a functional cure for or eradication of HIV-1, has yet to be achieved because of the persistence of latent HIV-1 reservoirs in adherent patients. The primary source of these viral reservoirs is integrated proviral DNA in CD4^+^ T cells and other non-T cells. Although a small fraction of this proviral DNA is replication-competent and contributes to viral rebound after the cessation of cART, >90% of latent viral reservoirs are replication-defective and some contain high rates of G-to-A mutations in proviral DNA. At least in part, these high rates of G-to-A mutations arise from the APOBEC3 (A3) family proteins of cytosine deaminases. A general model has shown that the HIV-1 virus infectivity factor (Vif) degrades A3 family proteins by proteasome-mediated pathways and inactivates their antiviral activities. However, Vif does not fully counteract the HIV-1 restriction activity of A3 family proteins *in vivo*, as indicated by observations of A3-mediated G-to-A hypermutation in the proviral DNA of HIV-1-infected patients. The frequency of A3-mediated hypermutation potentially contributes to slower HIV-1/AIDS disease progression and virus evolution including the emergence of cytotoxic T lymphocyte escape mutants. Therefore, combined with other strategies, the manipulation of A3-mediated mutagenesis may contribute to an HIV-1 functional cure aimed at cART-free remission. In this mini-review, we discuss the possibility of an HIV-1 functional cure arising from manipulation of A3 mutagenic activity.

## Introduction

Human immunodeficiency virus type 1 (HIV-1) was first discovered in 1983 as the agent that causes acquired immune deficiency syndrome (AIDS) (Barre-Sinoussi et al., 1983). HIV-1 disrupts the immune system by infecting immune cells, such as CD4^+^ T cells, macrophages, and dendritic cells, which ultimately leads to AIDS and related opportunistic infections. AIDS has become a manageable chronic disease due to the development of combination antiretroviral therapy (cART), which has enabled complete suppression of detectable viremia and controls disease progression in adherent patients. However, cART does not eliminate HIV-1 from these patients because latent reservoirs with the HIV-1 genome integrated into host DNA remain present (Chun et al., 1997; Finzi et al., 1997; Wong et al., 1997) and these latently infected cells clonally expand (Ikeda et al., 2007; Maldarelli et al., 2014; Wagner et al., 2014). When the latent reservoirs harbor replication-competent proviral DNA, they contribute to viral rebound when cART is interrupted (Chun et al., 1997; Finzi et al., 1997; Wong et al., 1997).

Efforts have been made to quantify the size of the latent reservoirs [reviewed by Wang et al. (2018); Lambrechts et al. (2020)]; studies have demonstrated that the majority of proviral DNA in the latent reservoirs is replication-defective and does not contribute to viral rebound when cART ceases. Ideally, complete eradication of HIV-1 from infected patients is the goal of a HIV-1 cure; however, because >90% of latent reservoirs harbor defective viruses, the initial goal should be a functional cure for HIV-1, which would comply with detectable proviral DNA and undetectable (or low-level) plasma viremia without cART. Of particular interest are elite controllers and long-term non-progressors, who represent <1% of all patients living with HIV-1 and are natural models for the functional cure [reviewed by Gonzalo-Gil et al. (2017); Promer and Karris (2018)]; however, little is known about the underlying mechanisms by which the immune systems of such patients control the virus.

**FIGURE 1:**
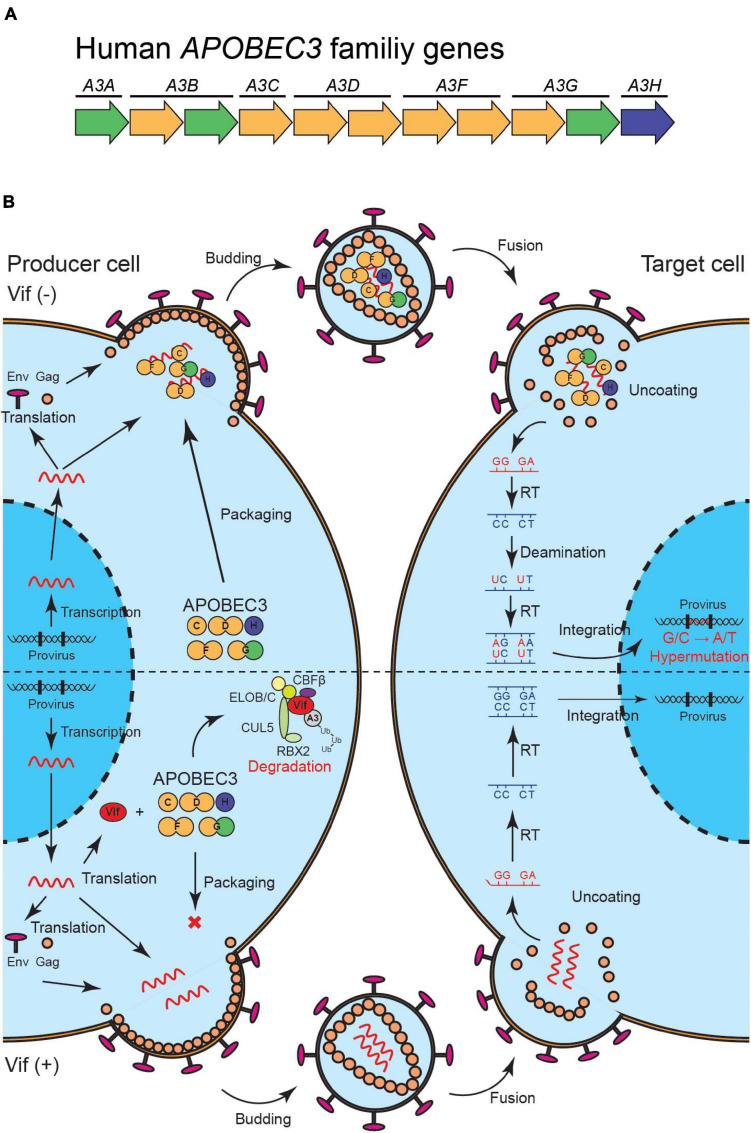
A model of HIV-1 restriction by A3 family proteins and counteraction by HIV-1 Vif. **(A)** Illustration of human *A3* family genes. Human *A3* family genes are composed of seven members with one or two zinc-coordinating domains (single of double-domain deaminases); these belong to three phylogenetically different groups, which are shown in green, yellow, and blue. **(B)** A schematic of HIV-1 restriction by five A3 family proteins and neutralization by HIV-1 Vif. A3C, A3D, A3F, A3G, and A3H are packaged into HIV-1 virions in producer cells and inactivate the virus through cytosine-to-uracil (C-to-U)/guanine-to-adenine (G-to-A) mutations **(top)**. HIV-1 Vif neutralizes the restriction activities of these A3 proteins through proteasome-mediated degradation **(bottom)**. Vif, virus infectivity factor; A3, APOBEC3.

**FIGURE 2:**
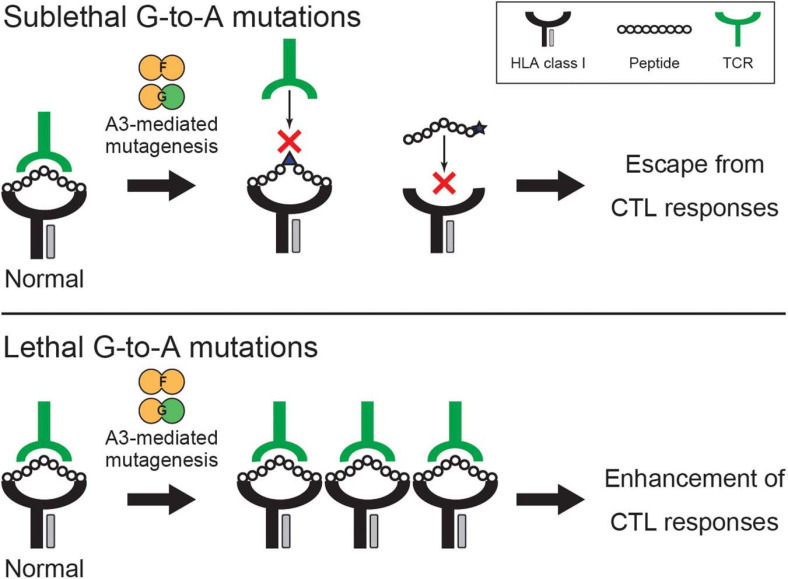
Potential effects of A3-mediated mutagenesis on CTL responses. A3F- and A3G-mediated mutagenesis alters CTL responses through the accumulation of G-to-A mutations on the viral genome, which leads to the modification of epitope sequences and their flanking regions involved in antigen processing/presentation, HLA binding, and TCR recognition. The two examples show that A3-mediated hypermutation on epitope sequences potentially alters HLA binding of the epitopes and TCR recognition. Sublethal A3-mediated mutagenesis is involved in the emergence of CTL escape variants **(top)**, whereas lethal A3-mediated hypermutation likely increases the number of HIV-derived epitopes and consequently enhances HIV-1-specific CTL responses **(bottom)**. A3, APOBEC3; CTL, cytotoxic T lymphocyte; HLA, human leukocyte antigen; TCR, T cell receptor.

APOBEC3 (A3) family proteins are cytosine deaminases that play important roles in mammalian innate immune responses. The human genome encodes seven *A3* genes on chromosome 22, which include three single-domain deaminase genes (*A3A*, *A3C*, and *A3H*) and four double-domain deaminase genes (*A3B*, *A3D*, *A3F*, and *A3G*) [reviewed by Koito and Ikeda (2012); Desimmie et al. (2014); Harris and Dudley (2015); Figure 1A]. In CD4^+^ T cells, up to five A3 proteins (A3C-Ile188, A3D, A3F, A3G, and A3H) are involved in HIV-1 restriction (Hultquist et al., 2011; Refsland et al., 2012, 2014; Ooms et al., 2013; Wittkopp et al., 2016; Anderson et al., 2018). These A3s are packaged into nascent viral particles and catalyze the deamination of cytosine-to-uracil in reverse transcription cDNA intermediates (Figure 1B) [reviewed by Harris and Dudley (2015); Simon et al. (2015)]. The uracil lesion provides a template for adenine insertion in HIV-1 genomic strands while accumulation of many G-to-A mutations ultimately leads to virus inactivation (Figure 1B). HIV-1 viral infectivity factor (Vif) recruits an E3 ubiquitin ligase complex to promote the degradation of A3 proteins through a proteasome-mediated pathway and counteracts their antiviral activity [reviewed by Harris and Dudley (2015); Salamango and Harris (2020); Figure 1B]. This model is well established and widely accepted. Nevertheless, reports have shown that some fraction of latent reservoirs harbors high rates of G-to-A mutations in proviral DNA (Kieffer et al., 2005; Ho et al., 2013; Imamichi et al., 2014; Bruner et al., 2016, 2019). Importantly, A3-mediated G-to-A hypermutation in proviral DNA is significantly correlated with disease progression in HIV-1-infected patients including elite controllers and long-term non-progressors (Pace et al., 2006; Land et al., 2008; Kourteva et al., 2012; Eyzaguirre et al., 2013; Cuevas et al., 2015). Additionally, CD4^+^ T cells from HIV-1 controllers with higher A3G expression levels are less susceptible to *in vitro* HIV-1 infection than are CD4^+^ T cells from HIV-1 controllers with lower A3G expression levels (Biasin et al., 2007; De Pasquale et al., 2013). Furthermore, A3-mediated mutagenesis seems to influence cytotoxic T lymphocyte (CTL) responses [reviewed by Stavrou and Ross (2015); Figure 2]. Taken together, these observations indicate that the mutagenic activity of A3 family proteins is a factor that determines HIV-1/AIDS disease progression. Therefore, the manipulation of A3-mediated mutagenesis to lethal levels may be a potential target for an HIV-1 functional cure. Here, we discuss the possibility of introducing an HIV-1 functional cure mediated by the mutagenesis of A3 family proteins.

## Contribution of *Apobec3* Gene Variations to HIV-1 Disease Progression

The *A3H* gene is likely a genetic factor that controls the disease progression of HIV-1 with the particular genotype of the *vif* gene. *A3H* is the most variable *A3* gene in the human population (Figure 1A). Four single-nucleotide polymorphisms and one indel in the *A3H* gene determine stable and unstable haplotypes of A3H proteins (OhAinle et al., 2008; Wang et al., 2011; Refsland et al., 2014; Ebrahimi et al., 2018). Stable A3H haplotypes are expressed stably and have the ability to inhibit HIV-1, whereas unstable A3H haplotypes are barely detectable or undetectable at the protein level and lack antiviral activity. Interestingly, not all HIV-1 Vif variants from laboratory and natural isolates can degrade stable A3H haplotypes. Several studies have identified amino acid residues at positions 39, 48, and 60–63 that influence the capability of Vif proteins to degrade stable A3H haplotypes but do not affect counteraction activity against A3D, A3F, and A3G (Binka et al., 2012; Ooms et al., 2013; Refsland et al., 2014). Hereafter, we denote Vif proteins that can degrade stable A3H haplotypes and other HIV-1-restrictive A3 proteins (i.e., A3D, A3F, and A3G) as “hyper-functional,” while we term those that fail to degrade stable A3H haplotypes but not other HIV-1 restrictive A3 proteins as “hypo-functional.”

Intriguingly, studies have shown that A3H haplotypes are correlated with the global distribution of HIV-1 Vif alleles (Refsland et al., 2014; Nakano et al., 2017b). For example, around 60% of people have stable A3H haplotypes in Sub-Saharan Africa where hyper-functional Vifs are dominant (Refsland et al., 2014; Nakano et al., 2017b). In contrast, hypo-functional Vifs are prevalent in Asia because most people have unstable A3H haplotypes (Refsland et al., 2014; Nakano et al., 2017b). This biogeographical relationship strongly suggests that HIV-1 with hyper-functional, too Vif has evolved to counteract stable A3H haplotypes but that stable A3H haplotypes remain a potential transmission barrier against HIV-1 with hypo-functional Vif outside Africa. This possibility is supported by previous studies showing that HIV-1-infected patients (including long-term non-progressors) with at least one allele of stable A3H haplotypes have slower disease progression to AIDS (Ooms et al., 2013; Sakurai et al., 2015). Therefore, stable A3H haplotypes may be effective for short-term HIV-1 suppression. However, before stable A3H haplotypes can be exploited for long-term HIV-1 suppression, further studies should ascertain the period over which they can control HIV-1 with the hypo-functional *vif* gene in the absence of cART and which variants emerge under the selective pressure of stable A3H haplotypes *in vivo*.

## Regulation of Apobec3 Family Proteins

Certain populations of proviral DNA in HIV-1-infected patients comprise defective viruses with A3-mediated G-to-A hypermutation (Kieffer et al., 2005; Ho et al., 2013; Imamichi et al., 2014; Bruner et al., 2016, 2019), suggesting that some of the A3 family proteins may avoid Vif-mediated neutralization *in vivo*. A simple explanation for this relates to the abundance of A3 family proteins, which can quantitatively exceed the capacity of Vif proteins (Figure 1B). In support of this explanation, CD4^+^ T cells from HIV-1 controllers with higher A3G expression levels were found to be more resistant to HIV-1 infection *in vitro* compared with CD4^+^ T cells from HIV-1 controllers with lower A3G expression levels (Biasin et al., 2007; De Pasquale et al., 2013). Therefore, the upregulation of A3 family proteins seems to increase defective viruses. However, the regulatory mechanisms of A3 family proteins have yet to be fully elucidated, largely because the expression of these proteins is intricately regulated and dependent on multiple determinants such as cell types (Koning et al., 2009, 2011; Refsland et al., 2010; Berger et al., 2011; Burns et al., 2013; Ikeda et al., 2019) and the surrounding environment of development and inflammation, e.g., the interferon (IFN) response (Peng et al., 2006, 2007; Pion et al., 2006; Ellery et al., 2007; Berger et al., 2011; Koning et al., 2011; Land et al., 2013; Mohanram et al., 2013).

Interestingly, most A3 family proteins are incorporated into ribonucleoprotein complexes in human cells (e.g., CD4^+^ T cells). In these cells, A3G forms enzymatically inactive high molecular mass (HMM) complexes immediately after translation, which are composed of A3G-binding proteins, A3G-binding RNAs, and RNA-binding proteins (Chiu et al., 2006; Soros et al., 2007; Stopak et al., 2007). These complexes are shifted to enzymatically active low molecular mass (LMM) complexes by RNase treatment (Soros et al., 2007). Hence, the formation of A3G HMM complexes in cells is one factor that determines permissiveness to HIV-1 infection (Pion et al., 2006; Ellery et al., 2007), being markedly altered by cytokines such as IFNs and chemokines (Kreisberg et al., 2006; Stopak et al., 2007). Similarly, the formation of HMM and LMM complexes in cells is conserved among other HIV-1 restrictive A3s (at least in A3F and A3H) (Niewiadomska et al., 2007; Ito et al., 2018). Although >100 A3G interactors have been identified (Chiu et al., 2006; Kozak et al., 2006; Gallois-Montbrun et al., 2007; Shirakawa et al., 2008; Sugiyama et al., 2011), *bona fide* interactors that regulate the restrictive capacity of A3s against HIV-1 are yet to be clearly defined. Accordingly, identifying novel A3 interactors that modulate the mutagenic activity of A3 enzymes against HIV-1 and resolving the molecular mechanisms that control these regulatory factors will be important for developing an HIV-1 functional cure that functions *via* A3-mediated mutagenesis.

## Potential Utilization of Vif-Resistant A3 Proteins for an HIV-1 Functional Cure

APOBEC3 proteins are examples of host factors that restrict cross-species transmission of lentiviruses [reviewed by Nakano et al. (2017a); Uriu et al. (2021)]. Interactions between mammalian A3 proteins and lentiviral Vifs are largely specific to viruses in their hosts (Mariani et al., 2003; Compton et al., 2012; Etienne et al., 2015; Yamada et al., 2016; Zhang et al., 2016; Nakano et al., 2020). As examples, HIV-1 Vif can degrade A3G protein from humans but not from African green monkeys or rhesus macaques (Bogerd et al., 2004; Schrofelbauer et al., 2004; Xu et al., 2004). Three laboratories have demonstrated that a single amino acid residue at position 128 of the A3G protein determines this interaction; human A3G D128K proteins, therefore, become resistant to HIV-1 Vif (Bogerd et al., 2004; Schrofelbauer et al., 2004; Xu et al., 2004). One potential strategy for controlling HIV-1 under cART-free conditions would be the use of Vif-resistant A3 proteins such as human A3G D128K proteins. Recently, Delviks-Frankenberry et al. (2019) took a novel approach to control HIV-1 using self-activating lentiviral vectors that deliver the human *A3G D128K* gene to target cells. T cell lines in which the human *A3G D128K* gene was transduced by this system were shown to potently inhibit HIV-1 replication and suppress the emergence of resistant viruses against human A3G D128K proteins for >3.5 months. The delivery system was also used to transduce primary CD4^+^ T cells as well as CD34^+^ hematopoietic stem and progenitor cells with transduction efficiencies of around 15 and 30%, respectively. Thus, developments in the efficiency of gene-of-interest delivery may improve the feasibility of gene therapy involving Vif-resistant A3 proteins as an HIV-1 functional cure.

Another interesting challenge is the creation of a “super restriction factor” to function as an inhibitor more potent than the original protein (McDonnell et al., 2020). Single-domain deaminase A3C proteins have weak HIV-1 restriction activity compared with that of double-domain deaminases, e.g., A3D, A3F, and A3G proteins (Bishop et al., 2004; Hultquist et al., 2011; Wittkopp et al., 2016; Anderson et al., 2018). Synthetic tandem double deaminase A3C proteins represent one attempt to create HIV-1 restrictive A3C proteins that are more potent than the native proteins (McDonnell et al., 2020). The novel double-domain A3C proteins showed improved HIV-1 restriction activity and were largely resistant to Vif-mediated degradation. Although an efficient delivery system to HIV-1 target cells must be established, a strategy by which single deaminases are genetically connected could generate deaminases that are more potent and Vif-resistant; such proteins could be exploited to develop an HIV-1 functional cure.

## Association of Apobec3-Mediated Mutagenesis With CTL Responses

Clearly, A3 family proteins contribute to innate immunity against retroviruses including HIV-1. In addition, there is evidence that A3-mediated mutagenesis is involved in adaptive immunity, such as CTL responses, through changes to proviral sequences (Figure 2). This can be validated because A3 family protein expression in antigen-presenting cells, e.g., macrophages, varies upon HIV-1 infection or cytokine stimulation with IFN (Peng et al., 2006, 2007; Pion et al., 2006; Biasin et al., 2007; Stopak et al., 2007; Land et al., 2008; Koning et al., 2009, 2011; Berger et al., 2011; Hultquist et al., 2011; Mohanram et al., 2013). Moreover, this process provides an additional layer to a potential HIV-1 functional cure by A3-mediated mutagenesis, i.e., *via* innate and adaptive immune responses.

Human immunodeficiency virus type 1-specific CTL responses and their human leukocyte antigen (HLA) restrictions are likely to be determinants of viral replication control in HIV-1-infected individuals [reviewed by Macatangay and Rinaldo (2015)]. However, mutations accumulate on the HIV-1 genome during infection and escape variants that can avoid HIV-1-specific CTL responses subsequently emerge. HIV-1 sequencing data from patients has indicated that A3-mediated G-to-A mutations (mainly A3F and A3G) are embedded within the predicted CTL epitopes and flanking regions, suggesting that sublethal levels of A3-mediated mutagenesis are involved in the emergence of CTL escape variants [reviewed by Grant and Larijani (2017); Venkatesan et al. (2018); Figure 2]. In contrast, one study in which the Vif-null virus was used revealed that A3G-induced mutations enhance HIV-1-specific CTL responses (Casartelli et al., 2010). Interestingly, engineered defective viruses with premature termination codons caused by A3G can enhance the abundance of HIV-1-derived epitopes, resulting in the activation of HIV-1-specific CTLs (Casartelli et al., 2010; Figure 2). The degradation of polypeptides from defective mRNAs is efficiently presented on major histocompatibility complex class I molecules (Trentini et al., 2020). Indeed, defective HIV-1 proviruses retain the ability to transcribe mRNAs and produce proteins that can be recognized by HIV-1-specific CTLs (Imamichi et al., 2016, 2020; Pollack et al., 2017). Therefore, lethal A3-mediated hypermutation may contribute to the generation of HIV-1 peptides supplied to the pool of antigens presented on major histocompatibility complex class I molecules and to the enhancement of CTL responses (Figure 2). Although effects on CTL responses seem to differ depending on lethal or sublethal levels of A3-mediated mutagenesis, A3-mediated mutagenesis undoubtedly contributes to the quantity and quality of HIV-specific CTL responses. For successful HIV-1 control without cART, A3 family proteins may be required to function alongside factors associated with adaptive immunity such as CTLs. Understanding the potential role of A3 proteins in innate and adaptive immunity would create new avenues of possibility for an HIV-1 functional cure.

## Targeting the Apobec3/VIF Axis to Manipulate Apobec3-Mediated Mutagenesis

Proteasomal degradation of HIV-1-restrictive A3s by Vif limits the packaging of these A3 enzymes into viral particles and thereby prevents A3-mediated hypermutation in the subsequent viral infection (Figure 1B). Nevertheless, evidence increasingly suggests that sublethal levels of A3-mediated mutagenesis contribute to virus evolution including drug resistance and immune escape [reviewed by Grant and Larijani (2017); Venkatesan et al. (2018)]. One possible therapeutic strategy would involve restoration of the lethal mutagenic activity of A3 family proteins by direct inhibition of Vif. Indeed, this viral protein is an attractive therapeutic target because it is not known to have mammalian homologs. Moreover, it is difficult for Vif-null HIV-1 to adapt to cells expressing full HIV-1 restrictive A3 proteins simultaneously (Haché et al., 2008; Albin et al., 2010; Ikeda et al., 2018).

Many attempts have been made to discover small molecules that target Vif proteins. Of these small molecules, RN-18 and its analogs are particularly well studied. RN-18 was discovered by screening 30,000 small molecules for the effects of Vif on fluorescence-tagged A3G signals (Nathans et al., 2008). RN-18 has been shown to exhibit A3G-dependent anti-HIV-1 activity in three non-permissive T cell lines (IC_50_ = 6 μM in H9); RN-18 treatment reduces the expression of cellular Vif and increases A3G expression levels, leading to the packaging of more A3G in viral particles. In addition, structure–activity relationship studies have revealed RN-18 analogs with improved potency in their antiviral activity relative to that of RN-18 (Ali et al., 2012; Mohammed et al., 2012, 2016; Zhou et al., 2017; Sharkey et al., 2019). For example, the compound 12c enhances the antiviral activity of A3G by >150-fold compared with the effect of RN-18 (Zhou et al., 2017). Furthermore, a recent cell culture study reported the emergence of a resistant virus against the RN-18 analog IMC15 and proposed a docking model of IMC15 bound to the Vif-A3G-E3 ligase complex (Sharkey et al., 2019). Along with RN-18 and its analogs, IMB-26/35, MM-1/2, and VEC-5 have been identified as lead compounds [reviewed by Olson et al. (2018)]. Overall, the development of small molecules that restore the mutagenic activity of A3 enzymes remains at an early stage. However, structural findings, such as the elucidation of the A3F–Vif interface (Hu et al., 2019), help to develop Vif antagonists that are more potent and can improve strategies for an HIV-1 functional cure.

## Conclusion

HIV-1 has adapted to human cells such as CD4^+^ T cells; consequently, restriction factors, including A3 family proteins, are less effective against the virus. However, certain populations of HIV-1 are still exposed to lethal or sublethal A3-mediated mutagenesis, suggesting that the manipulation of this process is an attractive target through which to develop a functional cure for HIV-1. Therefore, in combination with several other strategies or therapies, A3-mediated mutagenesis could potentially be applied to functionally cure HIV-1.

## Author Contributions

TI drafted the manuscript. All authors edited, contributed to the manuscript, and approved the submitted version.

## Conflict of Interest

The reviewer DS declared a past co-authorship with the author TI to the handling editor. The remaining authors declare that the research was conducted in the absence of any commercial or financial relationships that could be construed as a potential conflict of interest.
